# The performance gut: a key to optimizing performance in high-level athletes: a systematic scoping review

**DOI:** 10.3389/fspor.2025.1641923

**Published:** 2025-10-20

**Authors:** Junior Carlone, Attilio Parisi, Alessio Fasano

**Affiliations:** ^1^ Department of Neurosciences, Biomedicine and Movement, University of Verona, Verona, Italy; ^2^ Department of Movement, Human and Health Sciences, University of Rome “Foro Italico”, Rome, Italy; ^3^ Division of Pediatric Gastroenterology and Nutrition, Department of Pediatrics, Massachusetts General Hospital for Children and Harvard Medical School, Boston, MA, United States; ^4^ Department of Nutrition, Harvard T.H. Chan School of Public Health, Boston, MA, United States; ^5^ European Biomedical Research Institute of Salerno, Salerno, Italy

**Keywords:** gut microbiome, gut microbiota, athletic performance, high-level athletes, sports training, sports nutrition

## Abstract

**Introduction:**

The gut microbiome represents a key ecosystem influencing athletic performance through energy metabolism modulation, inflammatory response regulation, and recovery optimization in high-level athletes. However, the relationship between performance and gut microbiome composition in high-level athletes remains poorly understood.

**Objectives:**

This systematic scoping review aims to map the current evidence on the relationship between training and gut microbiome in high-level athletes, identify specific patterns in microbial response to different training and sports, analyse the effects of nutritional interventions and highlight some methodological and knowledge gaps in the current literature.

**Methodology:**

Following the PRISMA-ScR framework, a systematic search was conducted on PubMed, Scopus and Web of Science (2015-2025). Studies were selected according to defined criteria, including a population of high-level athletes, interventions through training and/or nutritional protocols and based on outcomes related to performance and health.

**Results:**

Nineteen studies met the inclusion criteria, comprising 12 experimental studies and 7 systematic/narrative reviews. The analysis of the studies revealed possible sport-specific patterns in microbiome modulation, with distinctive alterations in metabolic profiles, significant correlations between microbial stability and athletic performance, synergistic effects between training and probiotic supplementation and significant impacts of nutritional strategies and hormonal contraceptives on microbiome composition. The heterogeneity in analysis methodologies and the limited duration of studies emerge as the main limitations of the present study.

**Conclusions:**

The evidence suggests that the significant role of the gut microbiome in athletic performance optimization may be considered in the future, highlighting the importance of implementing an integrated approach between training and nutrition. Further studies are needed to define specific microbiome trends for different types of sports, competition levels and supplementation targeted at implementing performance outcomes in high-level athletes.

**Systematic Review Registration:**

https://osf.io/yh49t, identifier YH49T.

## 1 Introduction

The gut microbiome is established from birth and is influenced by various factors, including the mode, time, and place of birth, the type of breastfeeding and weaning and the use of antibiotics (1–5). Some studies have confirmed that human milk oligosaccharides (HMO) positively modulate the newborn's microbiome through a prebiotic action, as does lactoferrin present in maternal serum (6–8). The structure of the intestinal microbiome remains stable in adults. Still, it differs between individuals, characterized by a specific enterotype, and during the last phase of life, it tends to undergo loss of bacterial diversity (9, 10). Additionally, it is primarily influenced by diet, particularly the amount of fibre intake, short-chain fatty acids (SCFAs) production, physical activity (PA), body composition and lifestyle (5, 11–16). The gut microbiome represents a complex ecosystem that influences human physiology through multiple mechanisms, including the modulation of energy metabolism, immune and inflammatory response and the production of bioactive metabolites (17–21). In high-level athletes, the role of the intestinal microbiome appears particularly relevant, emerging as a potential modulator of athletic performance and training adaptations. Still, in strenuous and prolonged training or training not managed appropriately, they can become harmful, generating states of chronic inflammation (12, 22–24). A properly balanced microbiome in sports activity can reduce inflammatory markers and the production of Reactive Oxygen Species (ROS), further attenuating macromolecule damage (25). The gut microbiome significantly impacts training, as suggested by a bidirectional relationship between PA and the microbial community (26). The understanding of this interaction, particularly in the context of high-level sports, is still incomplete today. A study conducted by Xu et al. highlights that elite athletes present distinctive taxonomic and functional profiles in their gut microbiome, with specific genera such as Clostridiales and Faecalibacterium being more prevalent compared to non-elite subjects (27). This finding was further supported by Barton et al., who observed that the gut microbiome of professional athletes differs significantly from that of sedentary individuals, particularly at the functional metabolic level (28). The variation in microbiota composition may also be influenced by intestinal permeability, a topic that has been thoroughly examined by Fasano, who emphasised the role of the zonulin protein in regulating intestinal tight junctions and how disruptions in this system can affect overall health and physical performance (29, 30).

Several studies have supported the metabolic advantages of specific microbiome profiles and specific intestinal bacteria. Scheiman et al. reported that elite runners possess a greater abundance of Veillonella (31). This genus has been shown to convert lactate produced during physical exercise into propionate, improving endurance performance such as running (31, 32). This is in line with the results of Manor et al., which indicate a positive correlation between Veillonella and vigorous PA, further supporting the notion that specific microbial populations can enhance athletic abilities (33). Moreover, the microbiome plays a crucial role in nutrient metabolism and immune function, which is particularly important for athletes engaged in high-intensity and strenuous training. Some authors illustrate the impact of altered intestinal permeability on the systemic inflammatory response, an important factor to consider for athletes experiencing significant physical stress (29, 30). The research conducted by Heimer et al. highlights the importance of immune modulation and gastrointestinal (GI) health in achieving optimal athletic performance (34). Furthermore, certain studies have shown that dietary choices can impact the microbiome. Murtaza et al. have pointed out that the nutritional practices of elite race walkers play a significant role in shaping their gut microbiota composition (22). This suggests that diet and physical exercise are crucial elements in modulating athletes’ microbiome and optimizing physical performance (35).

The microbiome possesses dynamic capabilities in response to training and dietary changes. Akazawa et al. found significant variations in the gut microbiota during different phases of training periodization among elite athletes (36). Research in elite volleyball athletes shows that gut microbiota maintains dynamic stability, adapting its composition in response to different phases of training, competition, and recovery periods (37). This underscores the importance of personalized, direct and indirect nutritional and training strategies to optimize microbiome composition to improve performance (38–42). Furthermore, Jäger et al. have indicated that probiotics could play a role in maintaining intestinal health and improving performance results, suggesting a potential intervention pathway in elite sports (32). However, the optimization of this dynamic relationship remains unexplored.

In summary, the gut microbiome of high-level athletes in homeostatic conditions is characterized by unique microbial profiles that can confer metabolic advantages, improve nutrient absorption, and support immune function through increased abundance of beneficial bacterial species, enhanced microbial diversity and superior efficiency of the immune system compared to the sedentary population (13, 28, 43, 44). Additionally, athletes might incur dysbiosis in overtraining and non-functional overreaching conditions. According to studies conducted on zonulin and intestinal permeability, inadequate stress could compromise the functionality of the intestinal barrier, creating a connection between overtraining, intestinal dysbiosis and potential performance decline (21). The interaction between structured physical exercise, diet and microbiome composition underscores the need for further research to explore targeted interventions that could optimize athletic performance through microbiome modulation and prevent possible states of alteration of the same.

## 2 Methods

This systematic scoping review was conducted following the PRISMA-ScR (Preferred Reporting Items for Systematic Reviews and Meta-Analyses extension for Scoping Reviews) methodological framework and registered with the Open Science Framework (OSF) under the following DOI: 10.17605/OSF.IO/YH49T. The protocol was developed *a priori* to systematically guide the research and analysis process (45).

### 2.1 Study design

The choice to conduct a systematic scoping review stems from the heterogeneity of methodological approaches in available studies, the complexity of the microbiome high-level athlete relationship, the need to map existing evidence, the importance of identifying significant gaps in current literature and the utility of synthesizing evidence to guide future research. The objectives of the systematic scoping review are to identify available evidence on the relationship between training and gut microbiome in high-level athletes with particular attention to performance related biomarkers. Furthermore, the review aims to identify recurring patterns in the microbiome response to various training modalities and intensities that may influence physical performance parameters, analyse direct correlations between specific microbial profiles and quantitative indicators of athletic performance such as power, endurance, recovery time, evaluate the effectiveness of nutritional interventions aimed at modulating the microbiome for the optimization of sports performance and highlight methodological and knowledge gaps that limit the understanding of the microbiome potential in current scientific literature.

### 2.2 Research strategy

The literature search was conducted on three databases PubMed, Scopus and Web of Science, from January 2015 to September 2025. The search string was developed using MeSH terms and keywords related to three main domains: intestinal microbiome, training/sports and athletic performance (“Gastrointestinal Microbiome” OR “Gut Microbiome” OR “Gut Microbiota” OR “Intestinal Microbiota”) AND (“Exercise” OR “Physical Activity” OR “Physical Activities” OR “Sports” OR “Athletic” OR “Athletics”) AND (“Training Program*” OR “Periodization” OR “Exercise Prescription” OR “Training Load”).

### 2.3 Eligibility criteria

The inclusion criteria were structured following the Population, Concept and Context (PCC) framework, including high-level or elite athletes (P), the gut microbiome, nutrition, and probiotics, prebiotics, postbiotics and symbiotic/postbiotic supplementation (C) and athletic performance (C). Eligible studies included original research, including randomized controlled trials (RCTs) and observational studies, as well as systematic and narrative reviews published in English language (46, 47). The exclusion criteria were equally precisely defined, including studies on non-high-level or non-elite athletes, studies with incomplete data on the microbiome, and studies on animal models only.

### 2.4 Study selection and data extraction

The selection of studies followed a three-phase process, initial screening of titles and abstracts, full-text evaluation of potentially eligible articles, and exclusion of duplicate articles. J.C. and A.F. conducted the selection process, with a third reviewer A.P. consulted to resolve any discrepancies. Data were extracted using a standardized form, including study characteristics, design, population, duration, microbiome analysis methodology, training protocols, nutritional interventions, primary and secondary outcomes, main results and limitations.

### 2.5 Data analysis

The analysis followed a narrative-descriptive approach, particularly identifying recurring patterns and methodological gaps. Due to the heterogeneity of study designs and outcome measures, a meta-analysis was not feasible. Data synthesis focused on thematic analysis and identification of convergent findings across studies.

### 2.6 Quality assessment

In accordance with scoping review methodology, no formal quality assessment tool was applied to individual studies, as the primary aim was to map the existing evidence and identify knowledge gaps rather than assess methodological quality (46, 47). However, study characteristics including design, population, methodology, and limitations were systematically recorded during data extraction to provide contextual information for interpretation of findings. For the 12 experimental studies included, we assessed key methodological characteristics including study design, sample size, presence of control groups, intervention duration and dropout rates (Supplementary Table S1). This evaluation revealed that 5 studies employed RCTs (48, 50, 52, 54, 55), 5 were observational/cross-sectional studies (17, 26, 36, 51, 53), 1 used a controlled intervention design (22) and 1 was a case study (49). Sample sizes ranged from 1 to 84 participants, with intervention durations varying from 7 days to 20 weeks. Key methodological challenges identified across studies included small sample sizes in most studies, short intervention durations in several studies and varied analytical approaches limiting direct comparisons. Additionally, conducting research with elite or high-level athletes may presents intrinsic logistical and ethical constraints, including limited participant availability, competition schedules and restrictions on experimental interventions during training and competition periods, which contribute to the observed methodological limitations alongside the inherent complexities of microbiome research methodologies.

## 3 Results

**Figure 1:**
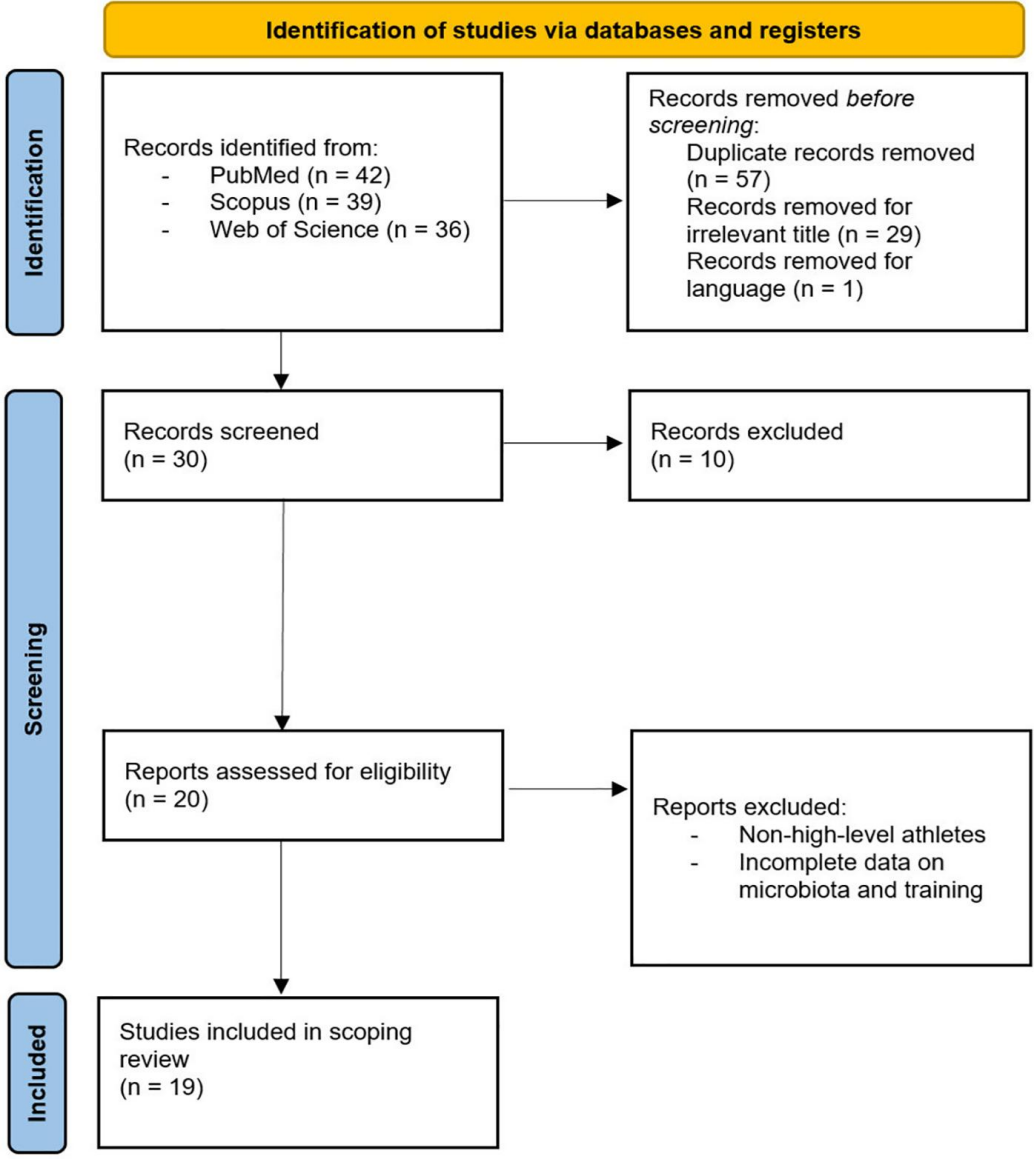
The PRISMA flow chart.

The research process initially identified 117 articles. After removing duplicates (*n* = 57), screening titles and abstracts (*n* = 29), and excluding (*n* = 1) non-English language article, (*n* = 30) articles underwent full-text screening. Of these, (*n* = 10) were excluded, leaving (*n* = 20) eligible articles. One additional article was excluded for focusing on non-elite athletes, resulting in a final inclusion of (*n* = 19) articles (Figure 1). Nineteen studies met the final inclusion criteria, comprising 12 experimental studies and 7 systematic/narrative reviews. The included experimental studies involved various athletic populations including Endurance sports (*n* = 4): Cyclists, Runners, Race Walkers, Mountain Trail Runner (22, 26, 48, 49), Power (*n* = 3): MMA athletes, Freestyle Wrestlers, Swimmers and Rowing (50–53), Team sports (*n* = 2): Basketball, Volleyball and Soccer (54, 55) and Mixed sports (*n* = 2): Multiple disciplines (17). The duration of interventions ranged from 4 to 20 weeks, with sample sizes between 16 and 84 participants and microbiome analysis methodologies primarily included 16S rRNA sequencing and Shotgun Metagenomics (Table 1). The systematic and narrative reviews provided analyses of the effects of probiotics (32, 34, 56), syntheses of adaptation mechanisms between the microbiome, performance and/or hormonal contraceptives (18, 57, 58) and an overview of practical applications (59) (Table 2).

**Figure 2:**
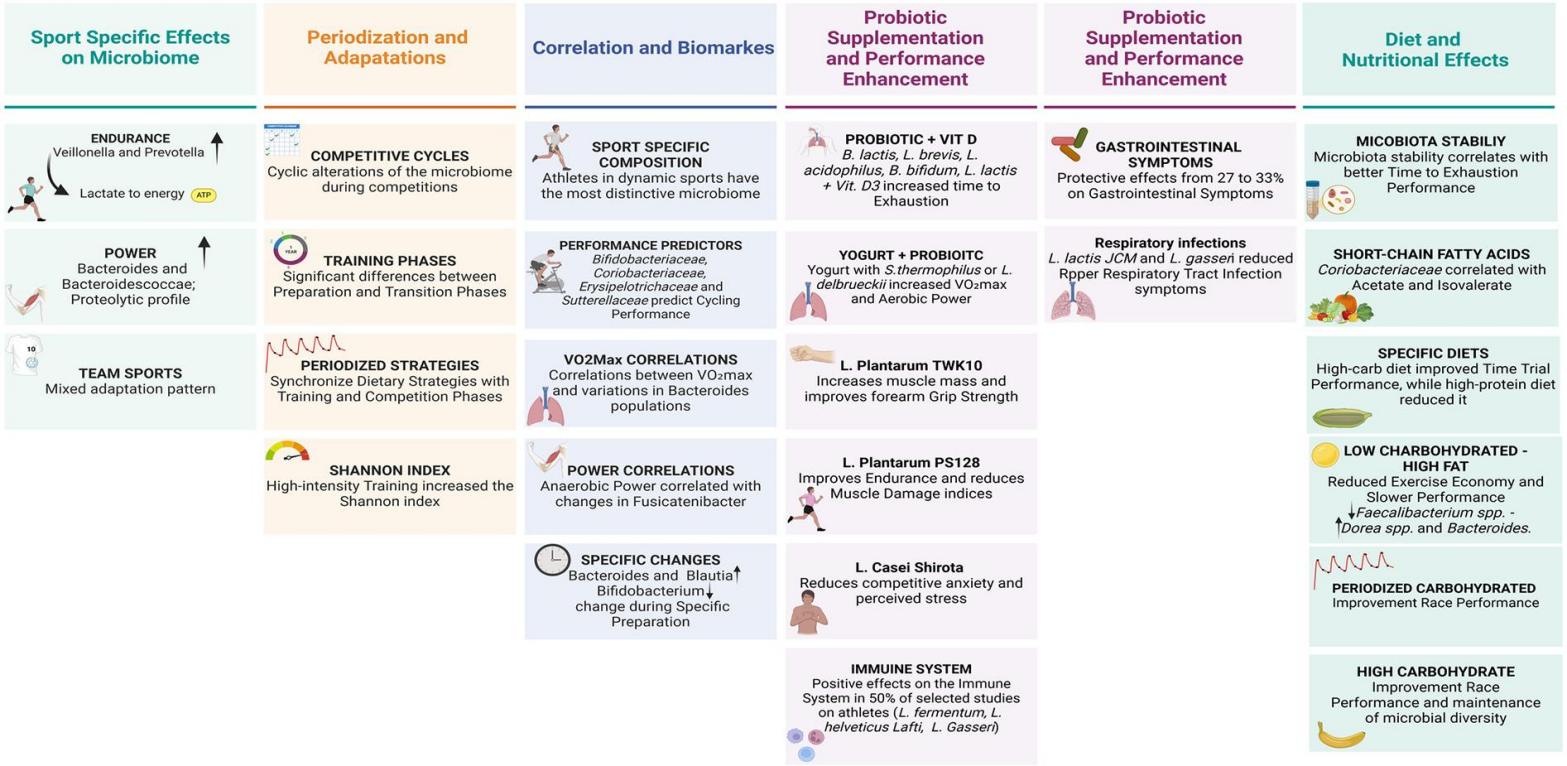
Sport-specific gut microbiome patterns and nutritional modulation in high-level athletes.

**Figure 3:**
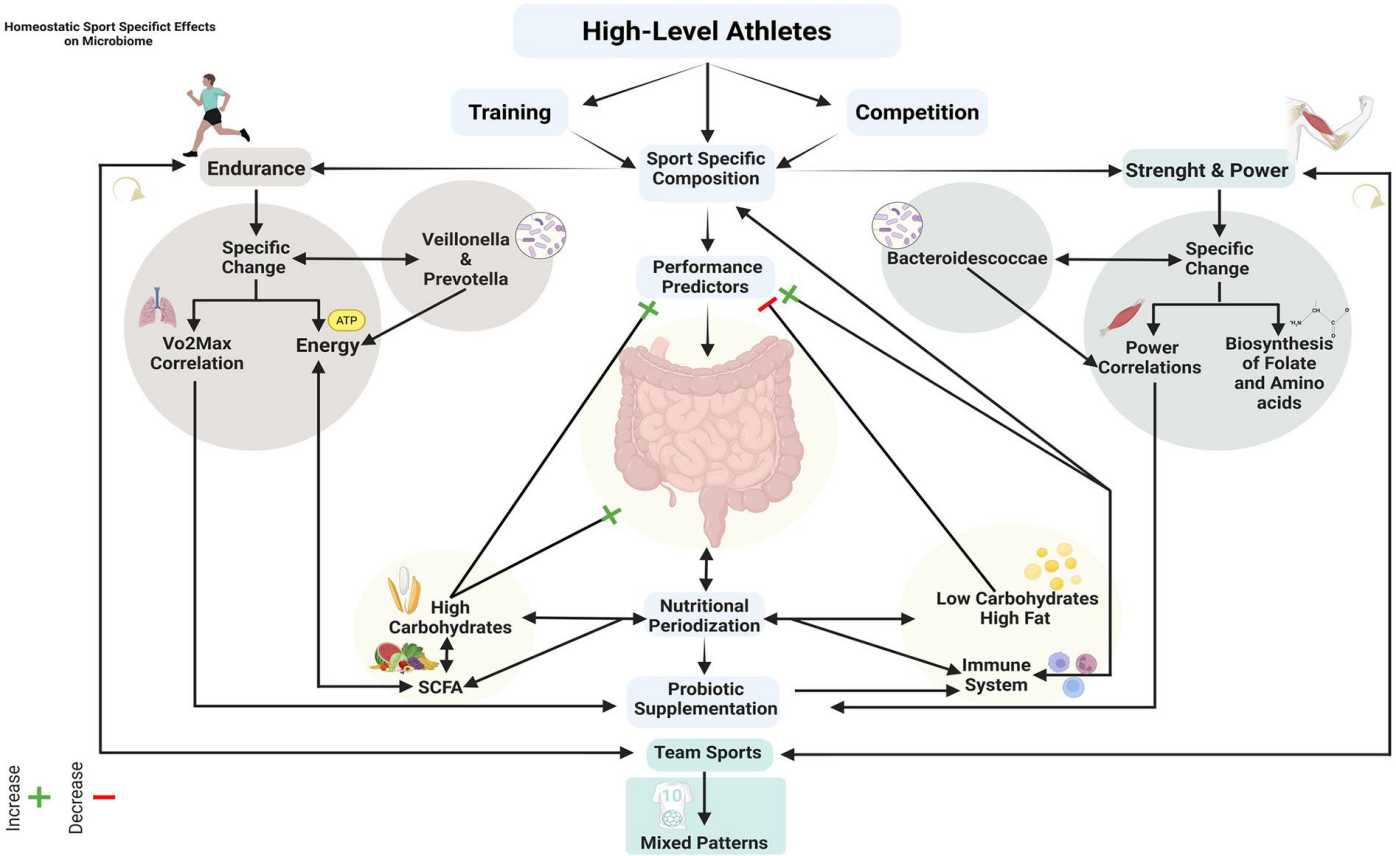
Integrated framework of training, nutrition and gut microbiome interactions in athletic performance optimization.

**Table 1:** Characteristics of included original studies.

Study	Population	Sample size	Duration	Methodology	Training type	Nutrition	Microbial activity	Metabolites	Key findings
Álvarez-Herms et al. (49)	Elite mountain trail runner	1 male (34 years, 171 cm, 59 kg, VO_2_max = 92 ml/min/kg)	5 month competitive season (6 samples)	16S rRNA sequencing	Mountain trail running, short races (42 km) vs. long races (172 km)	High CHO diet (CHO 54–63%; FAT 17–23%; PRO 20–22%); 4.1 L of fluid per day; 4,700 kcal/day; omega-3 and vitamin D3 and B12 supplementation	Season progression ↑Shannon diversity; post short race ↑Anaerotruncus, Butyricoccus, *Clostridium butyricum*, Lachnospira and Coprococcus; post long race ↑pathogenic bacteria Klebsiella, Citrobacter, Fusobacterium, *Salmonella enterica* and Shigella; ↓SCFAs producing bacteria	Post short race ↑Butyrate producing bacteria activity; post long race ↓SCFAs production and altered intestinal pH	↑Microbial diversity throughout season correlates with peak performance; short races promoted beneficial SCFAs producing bacteria; long races induced transient dysbiosis ↑Pathogenic bacteria; gut microbiota resilience supports recovery
Fu et al. (51)	Elite freestyle wrestlers	12 mixed (6 male and 6 females)	1 week (pre-competitio)	16S rRNA sequencing	Wrestling training (4× Heavy + 2× medium + 1× light load/week)	Optimal control weight group (<2 kg) ↓CHO and FAT, ↑PRO relative intake; Non Optimal Control Group (>2 kg) ↑CHO and FAT, ↓Protein relative intake	Optimal control weight group (<2 kg) ↑Solobacterium, Rothia, Fusicatenibacter, Abiotrophia, Brucella, Streptococcus; Non Optimal Control Group (>2 kg) ↑Christensenellaceae, Oscillospiraceae, *Eubacterium siraeum*, Lachnospiraceae; ↑^3^Phylogenetic Diversity in Non Optimal Control Group (>2 kg)	Differential metabolites (371) ↑141 and ↓230 in Optimal Control Weight Group (<2 kg) vs. Non Optimal Control Group (>2 kg); Key metabolites ↓Correlated with CHO Intake Cholic acid, Clinofibrate, Angiotensinamide, Prostaglandin J2; Pathways ↓Linoleic acid metabolism and ↑Tryptophan metabolism	Optimal control weight group (<2 kg) correlated with balanced dietary patterns, better training adaptation and distinct microbiota and metabolite profiles; Non Optimal Control Group (>2 kg) showed signs of inadequate adaptation
Charlesson et al. (53)	Elite rowing athletes	23 mixed (11 male and 12 female) 19 completed (7 male and 12 female)	3 day periods high training and low training; separated by 1 month	16S rRNA sequencing	Rowing, indoor rowing, cycling during high training vs. low training	Ad libitum intake; Athlete diet index score higher in high training vs. low training	High training ↑bacteroidetes, ↓Shannon diversity, ↓firmicutes/bacteroidetes ratio; stable enterotypes (prevotella vs. bacteroides dominant)	High Training ↑Total SCFAs, ↑Propionic acid and ↑Butyric acid	Training load influences gut microbiota composition and SCFAs production; Higher training associated with ↑SCFAs, altered bacterial abundance and ↑Stool frequency; Diet quality explains 12.2% of microbial variation during High Training
Fernandez-Sanjurjo et al. (26)	Elite cyclists athletes	16 male (15 completed)	3 weeks (grand tour)	16S rRNA sequencing	Professional cycling race with 4 sampling points (Pre-race, after 9 stages, after 15 stages and after 20 stages)	↑CHO intake during competition; ↑Sports supplements (drinks, gels, bars)	↑Bifidobacteriaceae, ↑Coriobacteriaceae, ↑Erysipelotrichaceae and ↑Sutterellaceae	SCFAs correlated with specific taxa Coriobacteriaceae with Acetate and Isovalerate, Bifidobacteriaceae with Isobutyrate	High correlation between microbial composition and final performance; Dietary components influence microbiome ↑CHO and ↓Bifidobacteriaceae
Akazawa et al. (36)	Elite athletes (cross-sectional) and elite short-track speed skaters (longitudinal)	Cross-sectional 84 athletes (35 female, 49 male) and longitudinal 10 athletes (6 female, 4 male)	Cross-sectional: single time point. Longitudinal: 3 months between General and Specific preparation phase	16S rRNA Sequencing	Training periodization during Transition or Preparation season and Sport-specific training	Normal dietary intake monitored	Cross-sectional ↓*Prevotella*, ↑*Bifidobacterium,* Parabacteroides and Alistipes in preparation vs. transition period; Longitudinal ↓Bacteroides, ↑Blautia and Bifidobacterium during Specific preparation	N/A	Training periodization alters gut microbiome abundance related to energy metabolism; Changes in specific bacteria correlated with aerobic capacity and anaerobic power; Microbiota stability associated with ↑Athletic performance
Kang et al. (54)	High-level basketball players	30 male (15 test group and 15 control group)	20 weeks	16S rRNA sequencing	Test group 24-style simplified Tai Chi 6 times/week, 90 min/session, at 70% max heart rate; control group regular routine	Normal dietary intake monitored	Test group showed ↑α-diversity index, ↑Blautia, ↓Proteobacteria abundance; Changes in Ruminococcaceae, Lachnospiraceae, Rikenellaceae, Prevotella, Faecalibacterium and Bacteroides	Not directly measured; observed changes in bacteria associated with Butyrate production	Tai Chi enhanced gut microbiota diversity ↓Harmful bacteria, ↑Blood lipid profiles and Blood pressure
Przewłócka et al. (50)	High-level MMA athletes	25 male (23 completed, probiotics + vitamin D3; vitamin D3)	4 weeks	Shotgun metagenomics	High-intensity training	N/A	↑β-diversity, Bacteroides, Roseburia, Prevotella, Negativicutes; ↓Lachnospiraceae, Peptostreptococcaceae and Calprotectin	No significant change	↑Aerobic performance and microbiota composition with Multistrain probiotic + Vitamin D3
Bielik et al. (52)	Elite swimmers athletes	24 mixed (17 male and 7 female)	7 weeks	16S rRNA sequencing	High intensity training with two groups HIT and HITB	HIT normal diet HITB cheese + probiotic for 3–4x/week	↑α-diversity in both groups, ↑Lactococcus in HITB vs. HIT, ↑Butyricimonas and Alistipes in HIT	↑Lactate and Pyruvate ↓Acetate and Butyrate	High intensity training ↑Gut microbiota diversity, regardless of probiotics
Furber et al. (48)	High-level endurance runners athletes	16 male (8 HCD group and 8 HPD group)	7 days	16S rRNA sequencing	Habitual endurance training	HCD 60% CHO, 10% PRO, 30% FAT vs. HPD 30% CHO, 40% PRO, 30% FAT (isocaloric)	HCD ↑Ruminococcus and *Collinsella spp.*; HPD ↑Sk1virus and Leuconostoc bacteria; ↓α-diversity of inducible viruses	N/A	HCD ↑Endurance performance with stable gut microbiota; HPD destabilized gut microbiota, particularly viral communities. Greater microbial stability associated with better performance
O'Donovan et al. (17)	Elite mixed athletes	37 mixed (14 female and 23 male) (A3 judo = 3, B1/B2 fencing = 3, C1 field hockey =15, C2 swimming/running =12 and c3 rowing =7)	Cross-sectional	Shotgun metagenomics	Low endurance/high power A3; moderate endurance/power B1/2; high endurance/low power C1; high endurance/moderate power C2; high endurance/high power C3	No significant diet differences between the sports groups	Low Endurance/High Power N/A; Moderate Endurance/High Power ↑*Streptococcus suis,* ↑*Clostridium bolteae,* ↑*Anaerostipes hadrus;* high endurance/low power ↑*Bifidobacterium animalis,* ↑*lactobacillus acidophilus;* high endurance/moderate power N/A; high endurance/high power ↑*Bacteroides caccae*	Low endurance/high power ↓Faecal creatinine vs. C1; moderate endurance/high power no significant differences; high endurance/low power ↑Faecal creatinine vs. other groups, ↓urinary lactate vs. C2/C3, ↓*Cis-aconitate* and *succinic acid* vs. C2; High Endurance/Moderate Power ↑*Cis-aconitate* and *succinic acid* vs. C1, ↑Lactate vs. C1; High Endurance/High Power ↑Lactate vs. C1, ↓Faecal creatinine vs. C1	Microbiome and metabolome profiles varied by endurance/power sport type independent of diet
Toohey et al. (55)	High-level mixed athletes (10 soccer and 13 volleyball)	23 female (11 probiotic and 12 placebo)	10 weeks	N/A	Resistance training 3–4 workouts/week and Sport-specific training	Recovery drink (45g CHO, 20g PRO, 2g FAT) + Probiotic (DE111, 5B CFU/day) or Placebo	N/A	N/A	Probiotic group showed greater ↓Body fat compared to placebo; both groups ↑Strength (squat, deadlift, bench press) and Vertical jump performance
Murtaza et al. (22)	Elite race walkers	21 male (9 HCHO, 10 PCHO and 10 LCHF)	3 weeks	16S rRNA sequencing	Intensified training during training camps	Three diets HCHO 60% CHO, 16% PRO, 20% FAT/day; PCHO same macro/day split but periodized; LCHF 78% FAT, 17% PRO, <50 g CHO/day	Distinct enterotypes at baseline Prevotella or Bacteroides dominant; LCHF diet led to ↑Bacteroides and Dorea, ↓Faecalibacterium. HCHO/PCHO had small changes in Firmicutes	N/A	LCHF ↓Performance and impaired exercise economy despite increased fat oxidation; HCHO/PCHO ↑Race performance with minimal microbiota alterations; Athletes enterotypes remained stable despite dietary changes

CHO, carbohydrate; PRO, protein; L, liters; SCFAs = short-chain fatty acids; N/A, not applicable; MMA = mixed martial arts; HIT, high-intensity training; HITB, high-intensity training + probiotic cheese; HCD, high carbohydrate diet; HPD, high protein diet; CFU, colony forming units; HCHO, high carbohydrate diet; PCHO, periodised carbohydrate diet; LCHF, low carbohydrate high fat diet; ↑, increase/improvement; ↓, decrease/decline.

**Table 2:** Characteristics of included reviews.

Study	Type of review	Objective	Studies included	Population/setting	Methodology	Duration range	Intervention type	Key outcomes	Main findings	Limitations
Pierudzka et al. (58)	Narrative review	To examine the relationship between hormonal contraceptives, gut microbiota and exercise adaptation in female athletes	88 articles	Female athletes, physically active female and female	Narrative review	N/A	Hormonal contraceptives (combined oral contraceptives, progestin only methods) and Exercise interventions	Gut microbiota composition, exercise adaptation, SCFAs production and athletic performance	Hormonal contraceptives ↓SCFAs producing bacteria; Exercise may mitigate Hormonal contraceptives induced microbial disruptions	Limited direct evidence in athletic populations; single pilot study specifically in athletes
Cheng et al. (18)	Narrative review	To examine the relationship between athletes gut microbiota and performance optimization	Not specified in provided documents	Elite athletes (rugby, e-sports, endurance) and amateur athletes	Narrative review	N/A	Descriptive review	Gut microbiota composition, athletic performance	Significant differences in microbiota between elite and amateur athletes; Protein consumption associated with increased microbial diversity	Methodological differences across analysed studies, variability in sample, variability in training regimens, history, physical condition, environment, dietary intake affecting study outcomes preparation and sequencing
Teglas and Radak (56)	Narrative review	To summarize recent evidence on exercise-induced microbiota changes and evaluate probiotic supplementation on athletic performance across exercise modalities	N/A	Mixed athletes endurance (cyclists, runners, skiers); team sports (badminton, basketball, soccer)	Narrative review	4–20 weeks	Probiotics (single and multi-strain) Exercise training protocols	Athletic performance, cognitive functions, sleep quality, GI symptoms, URTI symptoms, inflammatory responses and body composition	Probiotics show strain and duration specific effects in both endurance and intermittent exercise sports. In Endurance sports Probiotics ↑Lipid metabolites, modulated VO_2_max and ↓GI symptoms. In Team sports Probiotics ↓Inflammatory activity and stress-related factors	Methodological variability across studies; need for standardized protocols and strain-specific interventions
Yang et al. (59)	Narrative review	To explore effects and mechanisms of microecologics on sports performance and post-exercise recovery	N/A	Elite athletes (swimmers, runners, triathletes, team sports) and murine models	Narrative review	1–16 weeks	Probiotics, Prebiotics, Postbiotics and Symbiotics	Sports performance, post-exercise recovery	Probiotics *L. plantarum* PS128, *Lactobacillus casei shirota*, *Bifidobacterium* BB-12; ↑Endurance, Muscle strength; ↓Damage markers and lowered anxiety	Scarcity of reviews on microecological effects, need for more high quality human trials
Kuibida et al. (57)	Narrative review	To identify mechanisms of interrelation between gut microbiota and physical loading	N/A	Endurance and sprint athletes	Narrative review	N/A	Regular exercise	Microbiota diversity, intestinal barrier function	Moderate exercise ↑Microbiota diversity; ↑*Lactobacillus acidophilus* in endurance athletes; ↑Bacteroidescoccae in sprinters; ↑ Veillonella bacteria in Marathon runners convert lactate into energy substrates and boosting endurance but causing gut inflammation	Mechanisms not fully elucidated; literature based study, no clear tendencies for physical load influence
Heimer et al. (34)	Systematic review	To evaluate the effects of probiotic supplementation on URTI, GI symptoms, and immune function in athletes and physically active individuals	41 studies (31 RCTs, 7 crossover trials and 3 longitudinal studies)	2,189 participants, aged 14–65 years professional/amateur athletes, recreationally active individuals and healthy adults	Systematic review following PRISMA guidelines	<5 weeks to 20 weeks (mean 8 weeks)	*Lactobacillus* strains, *Bifidobacterium* strains, multi-strain probiotics	URTI and GI symptoms and immune function	Significant positive effects in 50% of studies for URTI and Immune function; 27–33% positive effects for GI symptoms in athletes; Heterogeneous outcomes across studies	Reporting and publication bias; recommendation to include serum markers like zonulin in future studies uncertainty due to self-reported data and need for standardized definitions
Jäger et al. (32)	Position stand/ narrative review	To provide an objective and critical review of mechanisms and applications of probiotics for optimizing health, performance and recovery in athletes	N/A	Mixed athletes (runners, cyclists, swimmers)	Position stand/narrative review	N/A	Various probiotic strains *L. fermentum* VRI-003, L. casei Shirota, *L. helveticus Lafti* L10 and multi-strain probiotics	Athletic performance, VO_2_max metrics and time to fatigue	Aerobic Capacity and VO₂ max ↑*S. thermophilus*/*L. bulgaricus* yogurt and multi-strain probiotic yogurt in female swimmers; Training Performance ↑Training load (multispecies blend); Endurance ↑*L. plantarum* TWK10 and ↑Time to fatigue (multi-strain probiotics); Recovery & Health ↓URTI symptoms and better fatigue recovery/mood with *L. fermentum*, *L. helveticus Lafti* and *L. gasseri*	Limited research specific to performance outcomes; mixed results across studies; need for well designed trials

N/A, not applicable; SCFAs, short-chain fatty acids; GI, gastrointestinal; URTI, upper respiratory tract infections; VO_2_max, maximal oxygen uptake; RCTs, randomized controlled trial; PRISMA, preferred reporting items for systematic reviews and meta-analyses; LCHF, low carbohydrate high fat diet; ↑, increase/improvement; ↓, decrease/decline.

The analysis of collected data highlights a significant relationship between the intestinal microbiome and athletic performance, suggesting that microbiome composition represents a determining factor for optimizing sports performance, but it is not yet fully understood. This relationship could manifest through multiple mechanisms, including modulation of energy metabolism, support for recovery processes, influence on the immune system and contribution to inflammation management. O'Donovan et al. have demonstrated that athletes present distinctive microbial compositions correlated with the type of sport practiced (17). Athletes of sports with high dynamic components possess different microbiome compositions compared to those who practice sports with both dynamic and static components (17). In particular, the correlation between specific bacterial taxa and performance parameters (17). Fernandez-Sanjurjo et al. have identified that bacterial families such as Bifidobacteriaceae, Coriobacteriaceae, Erysipelotrichaceae and Sutterellaceae are strong predictors of cycling performance, as they can have an action on inflammation management and SCFAs production (26).

### 3.1 Endurance sports

Endurance sports can increase Veillonella and Prevotella (22, 26). Kuibida et al. have highlighted that marathoners possess increased levels of Veillonella that convert lactate into energy substrates, enhancing aerobic resistance but can potentially incur states of intestinal inflammation (57). Moreover, O'Donovan et al. have highlighted that athletes of specialties such as race walking and prolonged running present high levels of *Lactobacillus acidophilus* (17). A detailed case study by Álvarez-Herms et al. provided interesting insights into the possible dynamic changes of the microbiota in an elite trail runner during an entire competitive season (49). The athlete presented maximal oxygen uptake (VO_2_max) levels of 92 ml/min/kg and showed a progressive increase in microbial diversity compared to peak performance (49). Furthermore, a possible distinction emerged between the effects of short (42 km) and long races (172 km) for the sport discipline, where 42 km races appeared to promote an increase in presumably beneficial bacteria and SCFAs producers, including Anaerotruncus, Butyricoccus, *Clostridium butyricum* and especially Lachnospira, Coprococcus, while 172 km races appeared to induce transient dysbiosis with increased opportunistic pathogenic bacteria including Klebsiella, Citrobacter, Fusobacterium, *Salmonella enterica* and Shigella and reduction of protective commensal bacteria (49). A positive association between microbial diversity and aerobic performance emerges from Przewłócka et al. data, which detected specific correlations between changes in VO_2_max and variations in Bacteroides populations. At the same time, anaerobic power was correlated with changes in Fusicatenibacter (50). Furthermore, training itself favors an increase in microbial diversity. Bielik et al. observed that high-intensity training increased the Shannon index regardless of probiotic supplementation (52).

### 3.2 Strength and power sports

Dedicated high-intensity training (HIT) in swimmers can generate an increase in α-diversity regardless of the use of probiotics (52). Furthermore, Kuibida et al. report that in athletes who practice power sports such as sprint specialties a prevalence of Bacteroidescoccae is observed (57). Additionally, in sports with mixed patterns between static and dynamic components, such as fencing, higher levels of *Anaerostipes hadrus* have been seen compared to other sports (17). Other sports with high static and high dynamic components, such as rowing, have shown pathways more expressed for folate and amino acid biosynthesis (17). Other authors, analyzing 12 elite freestyle wrestlers, highlighted how the effectiveness of pre-competition weight control may be correlated with distinct microbial patterns (51). Wrestlers with effective weight control, characterized by a difference from the target weight of less than 2 kg (<2 kg), were associated with low carbohydrate and high protein diets. Furthermore, they presented greater abundance of Solobacterium, Rothia, Fusicatenibacter, Abiotrophia, Brucella and Streptococcus, a more appropriate nutritional structure and greater adaptability to training compared to the group with less effective weight control, with a difference from the target weight greater than 2 kg (>2 kg) (51). The group with less effective weight control (>2 kg) instead showed higher levels of Christensenellaceae, Oscillospiraceae, *Eubacterium siraeum* and Lachnospiraceae, positively correlated with relative carbohydrate intake (51). Moreover, this group presented signs of possible inadequate adaptation to intensified training load, evidenced by the presence of leukocytes, occult blood and proteins in urine, suggesting a relationship between microbiota, metabolic adaptation and performance in wrestling (51).

### 3.3 Team sports

Team sports can generate a mixed pattern of microbiota adaptation. Interventions like Tai Chi could be a low-intensity strategy to integrate into training as a possible microbiota modulator, generating an increase in Bacteroidetes and a decrease in Proteobacteria (54). Moreover, team sports present high levels of dynamism, such as field hockey, which has been seen to have very high levels of *Lactobacillus acidophilus* (17).

### 3.4 SCFAs and energy metabolism

SCFAs produced by the gut microbiome emerge as key mediators in the relationship between microbial composition and athletic performance. Fernandez-Sanjurjo et al. have identified significant correlations between Coriobacteriaceae and acetate, between Coriobacteriaceae and isovalerate and between Bifidobacteriaceae and isobutyrate, highlighting the role of these bacterial metabolites in supporting energy metabolism during physical exercise (26). The same authors indicate that the relationship between microbiota and performance depends not on a single factor but on multiple factors.

The differential metabolomic analysis in the study by Fu et al. on 12 freestyle wrestlers identified 371 different metabolites between the group with effective weight control (<2 kg) and the group with ineffective weight control (>2 kg), with particular relevance for lipid and amino acid metabolism (51). Some key metabolites negatively correlated with carbohydrate intake included cholic acid, clofibrate, angiotensinamide and prostaglandin J2 (51). Functional enrichment revealed some specific patterns, including downregulation of linoleic acid metabolism and upregulation of tryptophan metabolism in the effective weight control group, suggesting a more favourable metabolic profile for inflammation management and energy regulation during the pre-competition phase (51).

### 3.5 Diet and performance

Diet is a determining factor influencing microbiota composition and consequently athletic performance. Furber et al. have demonstrated that microbiota stability is associated with better endurance performance (48). Specifically, a high-carbohydrate diet (HCD) was able to improve endurance performance, while a high-protein diet (HPD) had the opposite effect (48). Supporting this thesis, Sanjurjo et al. indicate that carbohydrates are key factors for the positive modulation of performance and the microbiota of athletes (26). Furthermore, Murtaza et al. have highlighted that a high-carbohydrate diet (HCHO) induced maintenance of microbial diversity and improvement in race performance, as did a diet with periodized carbohydrates (PCHO), concluding that a diet low in carbohydrates and high in fats (LCHF) induced negative alterations, including reduced exercise economy, slower race performance and a reduction of *Faecalibacterium spp.*, increase *Dorea spp.* and Bacteroides (22). Lastly, Jäger et al. reported that diet is a key modulator of the microbiota, where nutritional variations can generate a modification in the gut microbiota (32). Whey protein intake could positively influence microbial diversity, while greater consumption of carbohydrates and fiber in athletes is associated with an increase in Prevotella (32). The effect of fats on the microbiota of athletes is still not fully explored, their quality seems to have a determining role for athletes’ health (32).

### 3.6 Training and nutrition periodization

The periodization of training and nutrition can generate cyclic alterations of the microbiota during competitions and significant differences can occur between preparation and transition phases (26, 50). In swimmers, decreases in training volume combined with an increase in intensity can induce an increase in α-diversity (52). In line with these results, Akazawa et al. have highlighted that in Japanese elite athletes, during the transition phase, there was a significant decrease in the genera Bifidobacterium, Parabacteroides and Alistipes and an increase in Prevotella (36). Furthermore, they highlighted a significant reduction in the genus Bacteroides and an increase in Blautia and Bifidobacterium during the specific preparation phase, with changes correlated to modifications in VO₂max and anaerobic power (36). Other authors indicate a correlation between microbial stability and the optimization of physical performance (48). Some authors have shown that intensified training periodization can influence the gut microbiota, with variations partly associated with the effectiveness of weight control (51). The functional prediction of the study revealed that the microbiota of the group with less effective weight control was more enriched in riboflavin metabolism, as it could potentially play a compensatory mechanism to maintain GI health homeostasis under intensified stress (51). This suggests that the microbiota could serve as a possible biomarker for training load adaptability and effectiveness of weight control strategies in weight category sports (51). Charlesson et al. also introduce an often overlooked aspect in athlete microbiome research, the importance of gut transit time as a gut health marker (53). Their results show that low training load periods could involve slower transit times, evidenced by reduced evacuation frequency and a greater number of participants experiencing absence of bowel movements (53). Increased α-diversity, reduced SCFAs concentrations and reduced Bacteroides abundance were observed when transit times were slower (53).

### 3.7 Probiotics, performance, health and recovery

Some authors support the efficacy of mixed probiotic supplementation with *Bifidobacterium lactis*, *Levilactobacillus brevis*, *Lactobacillus acidophilus*, *Bifidobacterium bifidum* and *Lactococcus lactis*, as they have been seen to be able to improve various aspects of athletic performance associated with the use of Vitamin D3. Przewłócka et al. reported that the combination of probiotics and vitamin D3 increased the time to exhaustion during physical effort. In contrast, no significant changes were observed in the group with vitamin D3 alone (50). Jäger et al. reported a significant increase in VO_2_max and aerobic power with a yogurt supplementation containing *Streptococcus thermophilus* and *Lactobacillus delbrueckii subsp. bulgaricus* for a period of 30 days (32). They also reported increased endurance performance through *L. Plantarum* TWK10 and a better response to training load in athletes supplemented with a multispecies probiotic blend (32). Still, other studies have not found the same effects, so the authors indicate that the regulatory mechanisms of probiotics might have an indirect action, modulating other systems (32). Lastly, the authors suggest that supplementation with multi-strain probiotics is associated with improved aerobic performance, including increased VO₂max, aerobic power and time to exhaustion (32). Yang et al. have highlighted specific effects of various probiotic strains, *L. plantarum* TWK10 might be able to increase muscle mass more and improve forearm grip strength, *L. Plantarum* PS128 has been seen to be able to improve endurance performance and reduce indices of muscle damage and *L. casei Shirota* might be able to improve competitive anxiety and perceived stress (59).

In the field of strength and power, Toohey et al. analyzed the effects of *Bacillus subtilis* in female athletes, finding significant improvements in squat 1RM, deadlift 1RM, bench press 1RM and vertical jump (55). It is necessary to indicate that these improvements were similar to the placebo group, except for the significant reduction in body fat observed only in the probiotic group. This study observed no advantages of probiotic supplementation in increasing strength and power (55). The impact of probiotics on body composition parameters and athletic performance remains uncertain, requiring further studies to clarify the role of probiotics as ergogenic supplements (32).

A crucial aspect of athletic performance, especially in intense and prolonged training contexts, is the capacity for recovery and infection resistance. Heimer et al. have highlighted that probiotics have significant positive effects on the immune system in 50% of selected studies on athletes (34). Notably, endurance athletes showed the most significant reductions in pro-inflammatory factors following single probiotic intake (34). Confirming the theory, some authors report that a high training load can compromise the immune status of athletes, thus increasing the risk of upper respiratory tract infections (URTI) (32). Furthermore, the authors have highlighted that probiotic integration benefits the prevention of URTI (32). In high-level athletes, through probiotics such as *L. fermentum*, *L. helveticus Lafti* and *L. gasseri*, there was a decrease in symptoms of URTI and a more excellent state of health and recovery, changes not obtained through multi-strain probiotics (32). GI problems are prevalent in endurance athletes and can compromise physical performance and nutrient absorption, generating difficulties for athletes’ health. The authors have highlighted protective effects from 27 to 33% after probiotic intake on GI symptoms, but the topic is still controversial (34). Jäger et al. also indicate that studies on the effects of probiotics in athletes show contrasting results due to methodological variability (32). However, some research reports benefits through multi-strain probiotics with *B. bifidum* W23, *B. lactis* W51, *E. faecium* W54, *L. acidophilus* W22, *L. brevis* W63 and *L. lactis* W58 on reducing zonulin levels in high-level athletes, but further studies are needed regarding the reduction of GI symptoms and probiotic intake (32).

Teglas and Radak provided a detailed overview of probiotic effects in different sports, emphasizing how supplementation effectiveness depends on multiple factors, including specific strain, colony forming units (CFU), duration and frequency of intake (56). These factors can modulate the physiological impact of probiotics both independently from training protocols and in interaction with them, producing different effects. In endurance sports, probiotic supplementation appears to positively influence lipid metabolites, including SCFAs, modulate VO_2_max and improve exercise duration, while in sports characterized by intermittent exercise, probiotics may reduce inflammatory process activity and improve factors related to psychological stress, such as anxiety and depression (56).

In runners, administration of *Bifidobacterium animalis Lactis* and *Lactobacillus acidophilus* (10 × 10⁹ CFU for 30 days) appeared to reduce pro-inflammatory cytokine production and maintain CD8 + cell and effector memory cell populations (56). The use of *Pediococcus acidilactici* and *Lactobacillus plantarum* (3 × 10⁹ CFU for 4 weeks) did not produce effects on GI symptoms (56). *Bifidobacterium longum longum* OLP-01 (1.5 × 10¹⁰ CFU for 5 weeks) showed increased distance covered and greater intestinal microbiota abundance (56). *Bifidobacterium lactis*, *Lactobacillus brevis*, *Lactobacillus casei, Lactococcus lactis, Lactobacillus acidophilus*, *Bifidobacterium bifidum* and *Ligilactobacillus salivarius* (2.5 × 10⁹ CFU for 12 weeks) improved VO_2_max, 60 s ventilation, functional capacity, respiratory reserve and exercise capacity, also reducing GI symptoms (56). Finally, *Lactobacillus helveticus Lafti* L10 (5 × 10⁹ CFU for 6 weeks) appeared to reduce time to exhaustion in runners (56).

In road cyclists, supplementation with a multi-strain combination of *Lactobacillus* and *Bifidobacterium* (1 × 10¹¹ CFU for periods of 4, 12 and 16 weeks) showed increased aerobic capacity, VO_2_max, exercise duration to exhaustion and reduced heart rate (56). Similarly to cyclists, a combination of *Lactobacillus, Bifidobacterium, Enterococcus* and *Bacillus* strains for 90 days reduced GI symptoms without generating effects on VO_2_max and time to exhaustion (56). In skiing, *Bifidobacterium lactis* BL-99 (1 × 10⁹ CFU for 8 weeks) appeared to increase levels of SCFAs and polyunsaturated fatty acids, bile salts, knee extensor strength and VO_2_max (56).

In team sports, probiotic effects are equally varied. *Lactobacillus casei Shirota* (3 × 10¹⁰ CFU for 8 weeks) appeared to improve reaction time in cognitive tests of soccer players, while a blend of *Lactobacilli, Bifidobacteria* and *Streptococcus* strains (4.5 × 10¹¹ CFU for 4 weeks) showed no effects on VO_2_max (56). Moreover, *Lactobacillus casei*, *Bifidobacterium lactis* V9 and *Lactobacillus plantarum* P-8 (≥6–8 × 10⁹ CFU for 6 weeks) showed URTI reduction, increased Secretory Immunoglobulin A levels, decreased inflammatory factors, reduced maximum heart rate and lactate elimination rate (56). Administration of *Bifidobacterium lactis* CBP-001010, *Lactobacillus rhamnosus* CNCM I-4036, and Bifidobacterium longum ES1 (≥1 × 10⁹ CFU for 1 month) reported reduced stress, anxiety, and depression and increased post-exercise dopamine (56). Administration of *Lactobacillus casei Shirota* (3 × 10¹⁰ CFU for 6 weeks) in badminton players showed stress reduction and increased aerobic capacity, while Bifidobacteria and Lactobacilli strains (≥1.25 × 10¹⁰ CFU for 23 days) in basketball players appeared to show reduction of inflammatory processes and apoptosis of peripheral lymphocytes (56).

Pierudzka et al. highlighted an important gap in the scientific literature regarding the interaction between hormonal contraceptives, gut microbiota and exercise adaptations in female athletes (58). Only one pilot study appears to have directly investigated this relationship, showing that hormonal contraceptive use in physically active women appears to be associated with a reduction in SCFAs producing taxa, suggesting potential implications for energy metabolism and exercise adaptations (58). This complex interaction requires further research to develop personalized nutritional and training strategies for high-level female athletes using hormonal contraceptives.

## 4 Discussion

To date, we know that each athlete's gut microbiome possesses unique characteristics, and athletes in specific sports disciplines may exhibit similar trends. Moreover, the microbiome likely plays a role in optimizing physical performance. However, we still do not fully understand its effective impact on a large scale, nor the real differences in terms of its variation throughout a competitive year. The analysis of included studies has highlighted three fundamental aspects of the relationship between training and gut microbiome in athletes.

The results demonstrate distinctive microbiota adaptation patterns in relation to sport type and training load. Endurance athletes show a significant increase in Veillonella and Prevotella, which is associated with lactate metabolism and energy substrate utilization (31). In confirmation, O'Donovan et al. have demonstrated that sports with a high dynamic component generate metabolites such as lactate, succinic acid and cis-aconitic acid capable of generating specific patterns of the microbiome (17). In power sports, a prevalence of Bacteroides is observed, potentially correlated with more significant protein metabolism (52). These sport-specific adaptations suggest a functional plasticity of the microbiome in response to different metabolic demands (37).

### 4.1 Role of periodization

Training periodization emerges as a key factor in microbiome modulation. Longitudinal studies have highlighted cyclic alterations in microbial composition during different preparation phases, with significant variations between load and recovery periods (26, 50, 52). Charlesson et al. provided new considerations on the impact of training load on intestinal health markers in elite athletes (53). Their longitudinal study on elite rowers showed that high training load periods, compared to low training load periods, can modify SCFAs concentrations, reduce evacuation frequency, increase Bacteroides abundance and decrease α-diversity, with a lower Firmicutes/Bacteroides ratio (53). The authors also identified that training load independently influences microbiome composition even when controlling for changes in diet quality, evacuation frequency and sex (53). In particular, diet quality explains part of the microbial variation during high training load periods, while during low training load periods the training stress score appears to contribute more to the observable microbial variation (53) (Figure 2).

### 4.2 Interaction of nutrition, probiotics and microbiome

Nutritional interventions show a significant impact on microbiome composition and consequently on performance. Probiotic supplementation with multi-strains or single strains has been shown to generate benefits on aerobic physical performance and intestinal health, while on strength performance, at the moment, no significant benefits have been measured (32, 50, 55). High carbohydrate diet strategies favour microbial profiles associated with better endurance performance, while low carbohydrate diets can negatively alter the microbial ecosystem, as can high protein diets change the diversity of the microbiota in athletes (18, 22) (Figure 2).

### 4.3 Strengths and limitations

This systematic scoping review was conducted following the rigorous PRISMA-ScR methodological framework and was registered *a priori* with the Open Science Framework. The literature search was comprehensive, utilizing three major databases: PubMed, Scopus and Web of Science with a well defined search strategy. The inclusion of both experimental studies and systematic/narrative reviews provided a comprehensive overview of the current evidence. The narrative/descriptive analysis approach was appropriate for identifying patterns and knowledge gaps in this emerging field. Despite the robust methodology employed in this systematic scoping review, it is essential to highlight some significant methodological limitations that characterize this field of research. The variability in microbiome analysis methodologies across different studies limits the direct comparability of results, and the often reduced sample sizes of studies decrease the statistical power and generalizability of results (60). Furthermore, most studies have included male athletes. The interactions between diet, training, microbiome and performance make it difficult to isolate the specific contribution of single factors. The high variability in microbiome composition between individuals, moreover, represents a confounding factor that complicates the interpretation of results. The variability in intervention durations and training protocols is another limitation. Finally, there is an evident lack of standardization in outcome measures across studies.

### 4.4 Practical applications and future developments

Despite significant advances in gut microbiome research, science still faces the challenge of fully understanding the nuances of individualized responses to interventions, species redundancy, the importance of strain-specific variations, uncharacterizable microorganisms, host microbiome interactions and the long-term effects of microbial manipulations. Although these knowledge gaps, the collected evidence suggests several practical applications and directions for future research, but the fundamental pillar for microbiome modulation remains the nutritional approach (61). Yang et al. emphasize the need to consider individual differences in athletes and precision nutrition as a crucial element to optimize the effects of the microbiome (59). Probiotic supplementation is highly variable in effects but also essential and reasoning with personalized approaches with particular attention to strain specificity based on the athlete's individual profile, considering the sports discipline practiced, baseline microbiome characteristics, specific physical performance objectives and the athlete's health status, could in the future play a significant role in athletic performance. Some authors provide evidence supporting the need to synchronize dietary strategies with training and competition phases to maintain an optimal microbiota state (26, 48). Supporting these considerations, Jarret et al. report that current evidence indicates gut microbiome modulation by means of probiotic supplementation can have ergogenic benefits on endurance performance, while preliminary findings also suggest potential benefits for strength and power performance, although further research is required (62). The mechanisms are not fully understood, but may involve improved exercise recovery, immune function, nutrient absorption and alleviation of GI symptoms (62). These findings reinforce the importance of the previously mentioned synchronization approach, where it would be essential to consider diets according to the training period, adapting the nutritional approach to optimize microbial composition based on the specific needs of different phases of training periodization. The results support the utility of microbiome monitoring as a potential biomarker of training, recovery and health status in high-level athletes, integrating microbiome analysis in training periodization. Moreover, standardization in processual methodologies is necessary since, today it remains a very complex topic (63).

## 5 Conclusions

To date, we cannot yet claim to have a clear vision on the topic of microbiome and sport. This systematic scoping review provides a mapping of available evidence on the relationship between training and gut microbiome in high-level athletes. The analysis of included studies has allowed the identification of initial but not exhaustive recurring patterns in microbiome adaptations in response to training, highlighting specificities related to the type of sport and training load. The results support a significant but still premature role of the microbiome in optimizing athletic performance through sport-specific adaptations in microbial composition, modulation of energy metabolism and inflammatory response, interaction with nutritional interventions and probiotic supplementation and a correlation with performance and recovery markers. The gut microbiome emerges as a potentially determining factor for optimizing athletic performance and health, offering new possible perspectives for personalized interventions, as well as integrated nutrition and training strategies. To date, we know that the main modulator for microbiome composition is nutrition (Figure 3).

## Data Availability

The original contributions presented in the study are included in the article/Supplementary Material, further inquiries can be directed to the corresponding author.
