# Supplementary material for: The performance gut: a key to optimizing performance in high-level athletes: a systematic scoping review
Source: Front Sports Act Living. 2025 Oct 20;7:1641923. doi: 10.3389/fspor.2025.1641923 (PMC12580381; doi:10.3389/fspor.2025.1641923)
Supplement: Supplementary file 1 [file Table1.docx]

**The performance gut: sport-specific microbiome modulation in high-level athletes: a systematic scoping review**

**Table S1** Methodological Characteristics Assessment for Original Studies

| **Study** | **Study Design** | **Sample Size** | **Control Group** | **Duration** | **Dropout Rate** |
| --- | --- | --- | --- | --- | --- |
| Álvarez-Herms et al. (2025) | Case study | n=1 | No control | 5 Months | N/A |
| Fu et al. (2025) | Cross-sectional comparison | n=12 | Group comparison | 1 Week | Not reported |
| Charlesson et al. (2025) | Longitudinal observational | n=23 | Within-subject control | 3 Days/Period | Moderate (4 dropouts) |
| Fernandez-Sanjurjo et al. (2024) | Longitudinal observational | n=16 | No control | 3 Weeks | Low (1 dropout) |
| Akazawa et al. (2023) | Cross-sectional + longitudinal | Cross-sectional (n=84); Longitudinal (n=10) | N/A  +  Within-subject control | 3 Months | Not reported |
| Kang et al. (2023) | Randomized controlled trial | n=30 | Randomized control | 20 Weeks | Not reported |
| Przewłócka et al. (2023) | Randomized controlled trial | n=25 | Two intervention groups | 4 Weeks | Low (2 dropouts) |
| Bielik et al. (2022) | Randomized controlled trial | n=24 | Randomized control | 7 Weeks | Not reported |
| Furber et al. (2022) | Randomized controlled trial | n=20 | Dietary control | 7 Days | Moderate (4 dropouts) |
| O'Donovan et al. (2020) | Cross-sectional | n=37 | N/A | N/A | N/A |
| Toohey et al. (2020) | Randomized controlled trial | n=23 | Randomized control | 10 Weeks | Not reported |
| Murtaza et al. (2019) | Controlled intervention | n=21 | Active Control | 3 Weeks | Not reported |

*N/A = Not Applicable.*
